# Starting parenting in isolation a qualitative user-initiated study of parents’ experiences with hospitalization in Neonatal Intensive Care units during the COVID-19 pandemic

**DOI:** 10.1371/journal.pone.0258358

**Published:** 2021-10-29

**Authors:** Nina M. Kynø, Drude Fugelseth, Lina Merete Mæland Knudsen, Bente Silnes Tandberg

**Affiliations:** 1 Division of Paediatric and Adolescent Medicine, Department of Neonatal Intensive Care, Oslo University Hospital, Oslo, Norway; 2 Department of Nursing and Health Promotion, Acute and Critical Illness, Oslo Metropolitan University, Faculty of Health Sciences, Oslo, Norway; 3 Institute of Clinical Medicine, Faculty of Medicine, University of Oslo, Oslo, Norway; 4 The Norwegian Neonatal Network, Oslo University Hospital, Oslo, Norway; 5 Department of Paediatric and Adolescent Medicine, Drammen Hospital, Vestre Viken Hospital Trust, Drammen, Norway; 6 Lovisenberg Diaconal University College, Oslo, Norway; New Mexico State University, UNITED STATES

## Abstract

**Background:**

Worldwide, strict infection control measures including visitation regulations were implemented due to the COVID-19 pandemic at Neonatal Intensive Care Units (NICUs). These regulations gave restricted access for parents to their hospitalized infants. The consequence was limited ability to involve in the care of their infants. At Oslo University Hospital entry to NICU was denied to all except healthy mothers in March 2020. The absolute access ban for fathers lasted for 10 weeks. The aim of this study was to explore parental experiences with an infant hospitalized in the NICU during this absolute visitation ban period.

**Methods:**

We invited post discharge all parents of surviving infants that had been hospitalized for at least 14 days to participate. They were interviewed during autumn 2020 using an explorative semi-structured interview approach. Data were analyzed via inductive thematic analysis.

**Results:**

Nine mothers and four fathers participated. The COVID-19 regulations strongly impacted the parent’s experiences of their stay. The fathers’ limited access felt life-impacting. Parents struggled to become a family and raised their voices to be heard. Not being able to experience parenthood together led to emotional loneliness. The fathers struggled to learn how to care for their infant. The regulations might lead to a postponed attachment. On the other hand, of positive aspect the parents got some quietness. Being hospitalized during this first wave was experienced as exceptional and made parents seeking alliances by other parents. Social media was used to keep in contact with the outside world.

**Conclusions:**

The regulations had strong negative impact on parental experiences during the NICU hospitalization. The restriction to fathers’ access to the NICU acted as a significant obstacle to early infant-father bonding and led to loneliness and isolation by the mothers. Thus, these COVID-19 measures might have had adverse consequences for families.

## Introduction

At present, the entire world is still struggling with the COVID-19 pandemic. Social distancing is the most effective measure to maintain infection control until a sufficient percentage of the population have been immunized by disease or vaccination.

Knowledge on the intrauterine transmission of COVID-19 from an infected mother to her fetus is sparse; to date, there is no evidence of vertical transmission [1]. Nevertheless, it turns out that infants of COVID-19-positive, symptomatic mothers are more likely to be admitted to a NICU [2].

For parents, having an infant admitted to a NICU is even under normal conditions a stressful experience that may negatively affect the parents’ psychological wellbeing and may change the experience of the parental role [3, 4]. At the same time, this can create an imbalance in the existing family dynamics [5]. Infants have a fundamental right, as far as possible, to be cared for by his or her parents according to the UN Convention on the Right of the Child, Article 7 [6].

The infection control measures implemented during the COVID-19 pandemic separated infants from their families with expected short and long-term consequences for both the infants and their families [7]. International guidelines have presented conflicting recommendations on managing neonates and families in Neonatal Intensive Care Units (NICUs) during the pandemic. Isolation of mother and child is most commonly recommended in cases of suspected or confirmed COVID-19 infection. More individual approaches are used depending on the degree of the mother’s symptoms, satisfactorily available facilities (e.g. a single-family room and/or pressure-negative rooms), and available healthcare professionals with adequate personal protective equipment. The strict parental access policy, limiting the number of caregivers’ access to the unit from none up to only two, has been adopted by most hospitals [8].

These regulations had impact on the units’ family policies with restricted parents’ access to their infants and consequently their ability to involve in infants care. The strict regulations had decisive impact on the availability of therapy services and lactation consulting [9], resulting in adverse effect on breastfeeding and less time for parents to bond and participate in infant care [10, 11]. Considering long hospitalization in NICU, this adverse effect might potentially sustain beyond discharge.

Little is known about the short- and long-term consequences of such extreme restriction measures regarding infant health and development or parental bonding, attachment, and parenting. A recent study of 277 NICUs, investigated restrictions impact on parental presence in the NICU [9]. The study indicated that control measures introduced by the health care systems to prevent the spread of coronavirus, may have had substantial impact on parental and family well-being. However, the study design did not include in-depth interviews of the parents.

The first case of COVID-19 in Norway was registered on February 26, 2020, and the first hospital admission was in March 2020. On March 12, 2020, the Norwegian government implemented the strongest, most intrusive, and comprehensive infection control measures during peacetime in Norway.

The Norwegian Paediatric Association recommended that all mothers in NICUs should be encouraged to provide breastmilk via pumps; eventually, breastfeeding their infants according to the WHO recommendation of March 18^th^ [12].

At the Department of Neonatal Intensive Care, Oslo University Hospital, the largest tertiary center in Norway, strict infection control measures were implemented, with closure to all but healthy mothers. The restriction policy allowed mothers to visit their infant(s), participate in caregiving activities, provide skin-to-skin care, and breastfeed but were not allowed to use breast pumps in the unit. During each entrance into the unit, they had to pass strict access controls. They were strongly requested to comply with infection control measures applicable to the general population. Face masks were not obligatory. The restrictions were somewhat eased on May 29, 2020, when the units allowed the father/co-mother/partner/other caregiver (hereafter referred to as father) to visit the infant restricted to two evenings per week. Individual assessments were made in life-threatening situations. On June 26, 2020, the restrictions were further eased so that both parents could alternatively visit their child during individual visits. The latter regulation was still valid until mid-June 2021.

Absolute closure to fathers lasted for 10 weeks. Restrictions were debated nationwide in the news media after an interview with the parents of a preterm infant, where the father was not allowed to see his premature son until 5 weeks after birth [13]. In a column written following this interview, the father’s important role in the time after the birth of a critically ill newborn infant was highlighted. The parent’s right to have contact with their child as equal caregivers were emphasized. The strict infection control measures were criticized, stating that access restrictions could have major consequences for the family [14]. The Norwegian Association for Children with Congenital Heart Disease and the Norwegian Premature Association initiated this study to explore parents’ experiences with the strict infection control measures in NICU.

This study aimed to explore parental (mothers and fathers or other caregivers) experiences for families hospitalized with an infant in the NICU at Oslo University Hospital during the absolute visitation ban period due to the COVID-19 crisis.

Our research questions included:

- How did parents experience being hospitalized in the NICU during the COVID-19 restriction period?
- According to the parents, what consequences did the restrictions have on parent-infant attachment, parental role, and breastfeeding?

## Materials and methods

The study has a qualitative design with an explorative, semi-structured interview approach. This method is suitable when exploring human experiences [15]. The interviews had open-ended questions (S1 File) in order to provide rich, detailed information on the phenomena of interest [16]. The study is reported in accordance with the Consolidated Criteria for Reporting Qualitative Research (COREQ) [17] (S2 File).

### Setting

Under ordinary circumstances (i.e., non-pandemic conditions), parents have as a general rule unlimited access to stay with their infant in Norwegian NICUs. Skin-to-skin contact and breastfeeding are encouraged as early as the infant’s medical condition allows it. Health care insurance is publicly funded, hospital care is free of costs, and both parents are allowed full job-leave with compensation during hospitalization with their infant. Also, parents have 48 weeks of fully paid parental leave shared between them after discharge from the NICU. During NICU admission, parents live free of cost or at low cost either in the hospital’s hotel or at a parent accommodation, both in close vicinity to the NICU.

### Recruitment

We invited parents of survived infants who were admitted to the Department of Neonatal Intensive Care either directly after birth from the Delivery Department or after a stay at the Department of Cardiothoracic Surgery. The infants were hospitalized for at least 14 days during the most comprehensive restriction period and were discharged from the NICU at time of invitation.

Eligible informants were screened according to inclusion and exclusion criteria through the register in the Norwegian Neonatal Network [18]. A letter was sent to the eligible participants containing information about the study. Participants were informed about the researchers’ credentials, occupations, and clinical experience, as well as how the interviews were planned. Forty-seven families were eligible for inclusion and were approached. Parents responded to the researchers by E-mail or phone for more information or for acceptance to study participating. No reminders were sent.

### Participants

Nine mothers and four fathers responded positively and were interviewed. One of the families self-recruited as they had heard about the study and wanted to share their experience, even though they had not been hospitalized during the absolute visitation band for fathers. The interviewees represented nine families. In three of the interviews, both parents participated, otherwise mothers and fathers were interviewed separately. Three infants had one to two older siblings; seven had no siblings. The infants were hospitalized for mean (range) 59 (32–110) days. Their infants were born extremely preterm, very preterm or full-term.

### Data collection

Ten interviews were performed by the first (NMK) and last (BST) author together. None of the interviewers knew or had been involved in the care of their child during the stay. Four of the interviews were performed in a quiet room at a university campus, and six were performed through a secure web platform (Helsenet.no), owned by the Norwegian Ministry of Health and Care Services.

The parents were asked to tell freely about their hospital stay, their experiences of care and how they experienced preparations to transfer to home or to another hospital. By letting parents tell their story they thereby told what was important for them and what they emphasized. We used the interview guide as scaffolding and for structuring the interviews (S1 File). The interviews lasted between 29 and 65 minutes (mean 49 minutes). The interviews were recorded, and notes were written simultaneously throughout the interviews, followed by a summative discussion between the interviewers directly after the completion of each interview. Data saturation was not determined in advance of the study [19]. Braun & Clark [15 p. 50] suggest N = 6–10 interviews for a small project as this. Consequently, the number of participants and interviews complied with a suitable size of sample needed to demonstrate the patterns across the data set and were small enough to focus on the parents’ experiences and the research questions [15]. All parents who contacted the researchers to participate were included.

### Data analysis

We used thematic analysis with six phases, as described by Braun & Clark [15, 20]. The analysis was inductive, as we approached the data without strict theories.

All 10 interviews were recorded, transcribed verbatim, and checked for accuracy (NMK and BST). In the first phase, after listening to the interviews and discussing the notes, data corpus was read and re-read to *familiarize with the data*. Using medical (DF) and nursing (NMK, LMMK, BST) insights in neonatology, we profited from and were inspired by each other’s different angles, experiences, and expertise. *Initial codes* were generated individually by NMK and BST in the second phase. Further, the data and the initial codes were discussed and developed collaboratively. The codes identified interesting features and organized the data systematically into meaningful units related to our research questions. To obtain rigor, all themes and sub-themes were discussed by the authors until agreement. During the third phase, we *searched for themes* by organizing the codes into different themes with a higher level of abstraction. The *themes were reviewed* during phase four to assure that the themes would fit the coded data and entire dataset. The authors re-checked all quotations and the belongings sub- and overarching themes. This created a back- and forward process to increase trustworthiness. Quotations were used to emphasize the parents’ voice and the dependability of the results. In the fifth phase, we *defined and named* the themes that captured the essence of each of them. A scientific paper and report was written to the user organizations.

### Ethical considerations

The study was approved by the Oslo University Hospital’s review board and the hospital’s Data Protection Officer (certificate number 20/16594). All potential interviewees received written and oral information. They were informed about confidentiality, that their participation was voluntary, and that they could withdraw from the project at any time. All interviewees signed an informed consent form. Recorded interviews were deleted after transcription.

## Results

Nine mothers and four fathers were interviewed. The overall results confirmed that giving birth to a preterm and/or sick infant during the pandemic was uttermost demanding. Simultaneously, parents had to handle the COVID-19 restrictions, learn to know and bond with their newborn, and become a family.

We found three overarching themes: life-impacting COVID-19 regulations, exceptional times, and struggling to become a family, all of which possessed sub-themes. Especially the fathers’ limited access was experienced as life-impacting, and the parents had to raise their voice to be heard.

Being hospitalized during the first wave was experienced as exceptional and made parents seeking alliances by other parents in the same situation. Social media was widely used to keep in contact with the outside world. Despite this, the regulations also gave the parents some sort of quietness as the regulations kept too many visitors away.

**Fig 1:**
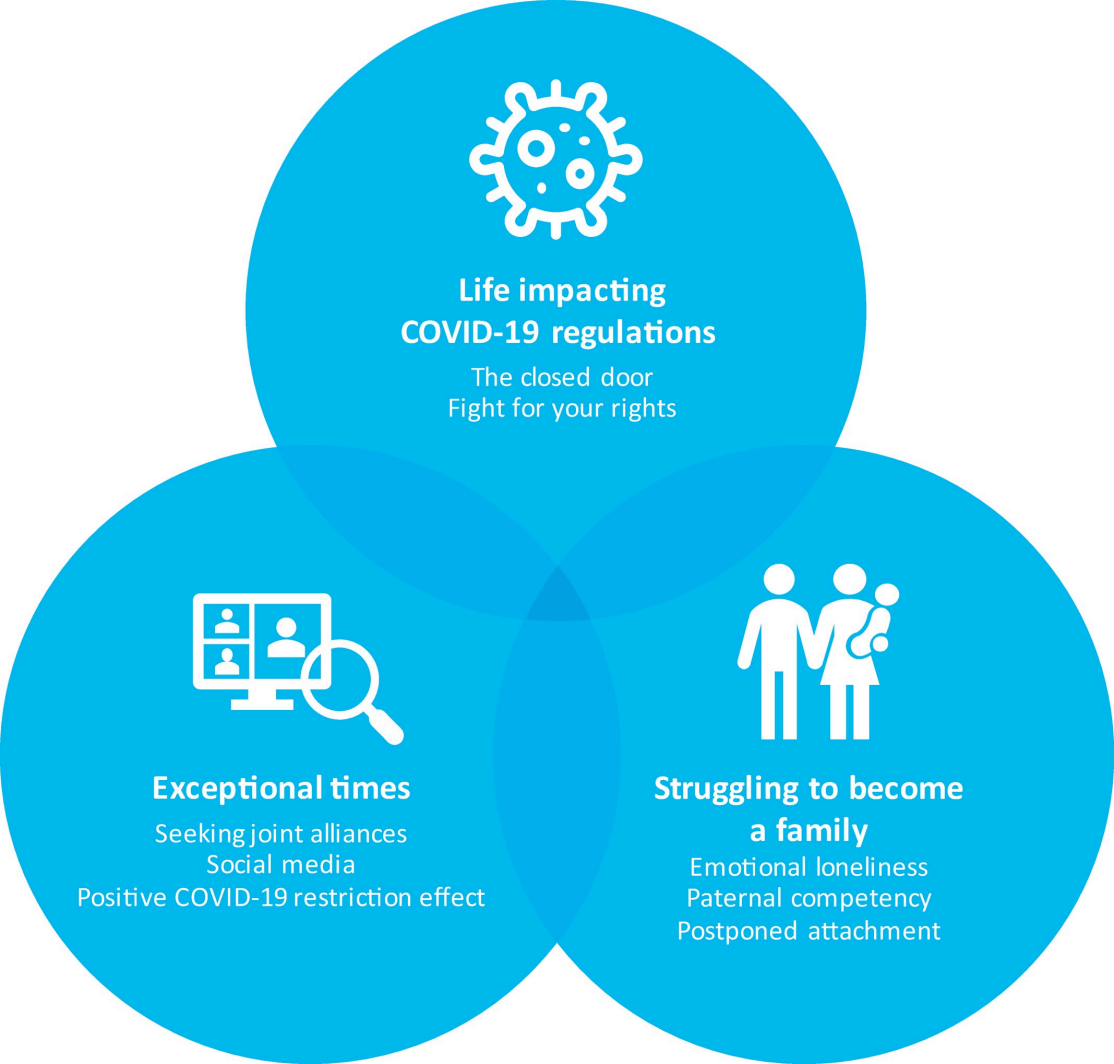
Map with the final overarching themes and the belonging sub-themes.

The parents struggled to become a family without being able to build parental relations through common experiences with their infant. Not being able to experience parenthood together led to an emotional loneliness. The fathers struggled to learn how to care for the sick and/or premature infant. The parents also told about the fear of how the regulations might lead to a postponed attachment. As one can see from the Fig 1, the themes are independent but to some degree interconnected.

### Life-impacting COVID-19 regulations

In the beginning, when social distancing was generally implemented in the society, the parents’ acceptance and understanding for the strict access policy due to the COVID-19 regulation was high,” … *of course*, *we understood the principles of reducing the number of people in the unit*, *we were at least as concerned as the staff …*.*”* (Mother T). The hospital appeared as a safe zone from a more dangerous unknown outside world.

*We immediately wondered how dangerous the COVID was for us …. but then we received information that children were not particularly exposed to risk; that was what we heard. Professionals were not worried, and this ease spread to us. We followed the nationwide news, but at the same time, we felt a little out of it all—in a separate bubble, thinking nowhere was safer than in the hospital… There were empty corridors in the hospital; people stayed away from each other for the first few weeks. I remember walking in the corridor, they were quite narrow, and I breathed in the other direction when I passed people… (laughs) It was quiet…. We had not been infected by passing… We knew nothing*. *(Mother B)*

As times goes by, the parents described an experience of dissatisfaction regarding the apparent novelty of the COVID-19 pandemic situation to both staff and leadership. *“They should have tried to coordinate [the information]*…. *There was a lot of divergent information*. *I wish they had written something down*… *I asked is there a place I can write*, *or a place I can read”* (Mother D). Parents reported a general lack of information. Especially the fathers tried to get information on what was going on with their infant and spouse inside the hospital … *“it would have been nice if I could only have 5 to 10 minutes with a doctor*, *who could have updated me instead of getting all the information from my wife who had so much else to think about”* (Father V).

#### The closed door

The consequences of the regulations were that the parents experienced the unit as nearly inaccessible, as if the door to the unit was out of reach for them, and especially the fathers.

In order to minimize exposure to the coronavirus by reducing the number of people in close contact, fathers’ access to the birth was restricted to solely the last sequence of the labor. One father described how the midwife urged him to go to the corridor straight after the birth so that he would be able to get a glimpse of his infant when passing by in the transport incubator.

The access restrictions were eased if the infant became critically ill and the situation was considered life-threatening. As soon as the infant regained a more stable medical condition, the fathers were again denied access. Some fathers did not see their infant for the first 9 weeks.

COVID-19 regulation hindered the mothers’ possibility to be present during medical rounds. Due to the risk of viral contamination, the use of headphones was prohibited. Additionally, the number of health professionals and parents in the patient rooms was restricted. Mothers reported a tight schedule; they would pump breastmilk eight times a day in their rooms, eat, sleep, wait for the medical rounds to finish, and then be with their infants as much as possible.

Most mothers brought up how COVID-19 restrictions directly affected pumping and caused significant added stress, both physically and mentally, in addition to preventing their access to their infant.

*…*. *because you could not pump in the room [with the infant]*, *then the time is limited*…. *When you have to go and cannot do it in the room; at the same time*, *you cannot visit [the infant] because it is a medical round and you are not allowed to take out [performing skin-to-skin] your infant*. *Also*, *you cannot come back because of that pumping*, *right*? *That pumping ruined the days there*, *I would say*…. *I think that was strict*. *Everyone had their own pump and their own chair*. *It had been completely unproblematic*. *Everyone had their own bag with equipment for the pump; it would not have changed anything if I had sat there*. *It would have made me able to be much more with the infant every day*. (Mother F)

After discharge, some mothers breastfed their infant exclusively and donated milk; others felt overwhelmed by the fact that they longed to go home and focused less on time to learn to breastfeed.

From the mid-May, fathers were permitted to visit their infants in the afternoon solely on Mondays and Thursdays and without the mothers. Twins were permitted with two parents. The families struggled to understand the rationale behind this decision.

*It was a bit unfair*, *and I did not fully understand the regulations*. *It makes very little sense that you can come Monday night and Thursday night*, *but not other times*.…. *There is an extremely large gap in the logic that fathers could visit mothers at the patient hotel and go to work—if I had COVID*, *she would take it further*. (Father V)

#### Fight for your rights

The nurses provided information to parents, and a common frustration related to the regulation evolved. Parents recounted how nurses tried to help them to speak up for their needs. Despite the nurse’s efforts, a lack of organization and leadership was questioned. Parents also questioned how and on what basis health personnel could impose restrictions on parents.

*It was the nurses who gave us information*, *but there was often a slight discrepancy between what the different nurses said*. *We wanted to get information from [a] person with authority*, *a leader perhaps*. *It is really serious when the father is not allowed to be present*, *more than we ask a random nurse and that says*: *“No*, *he is not allowed to come in*.*”* (Mother S)

The parents reported inconsistent enforcement of regulations resulting in frustration related to both the enforcement of the COVID-19 regulations and the overall lack of information. Mother R told: *“…*. *also there was a slight difference in personal attitudes between the nurses*, *what was okay [to do] and not*.*”* The parental confidence in the health personnel weathered by the wear and tear of living with the measures over time

*That is*, *they place a stronger burden on parents who are already under great pressure than they themselves are able to follow*, *and this is where it begins to break*, *that it is not logical*, *and it is not something they live by themselves—and there I think the management must come in and have a very clear tightening of the staff*, *where they clearly say what applies—personal opinions are not welcome*. (Mother T)

To compare restriction policies, parents contacted other NICUs for additional information. Some parents arranged meetings with the head of the Department to get more updated and thorough information. As mother S expressed: “*Well*, *that was a survival strategy*. *To really be on and turn every stone”* [to change the restrictions]. Despite questioning the rationale of the regulations, the mothers trusted the nurses and doctor’s medical expertise. However, mothers also believed that health personnel would attend to their need as parents, but discovered that it was "survival of the fittest" as regards:

*I have a little naively trusted that the hospital gives me the information I need… Then I realized afterwards that there were many moms who were much angrier than me and said much more; insisted much more*. *I did not because I thought…*…*for those who work there give me what I need*. *But they do not*. *If you don’t bother*, *you must in a way howl and scream to get enough attention*, *and I simply did not; I regret it a bit*. (Mother F)

### Exceptional times

Being hospitalized with a newborn infant in a NICU during a pandemic was described as a distinctive and portentous experience.

… how can it be described to outsiders how it feels to be in such a unit during a pandemic? It is very difficult. (Mother T) …. It’s intense—we’re moving in a “time in the time,” the bubble they’re talking about. That was how it was experienced because the sun went up and the sun went down, but we found no windows to get out. So, everything goes in slow motion, some kind of slow and crazy movie. (Father H)

Parents used different and multiple coping mechanisms to ease their extreme experiences and function in daily life while taking care of their families. Social media was widely used, but bonding with peers/other parents and health personnel became important coping strategies.

#### Seeking joint alliances

In lack of human contact because of the COVID-19 restrictions, mothers maintained contact both at the unit and at the parent accommodation. In addition, they connected with nurses who were with them all day. One mother (Mother Q) explained: *“They were so nice the nurses who sat and talked to me and realized that I just needed to talk to someone because I did not talk… I did not meet people ‘in person’ except at NICU*.*”* Mothers also commented that the nurses had to help them more now as their spouses could not.

Because of COVID-19, supportive parental measures, such as parents’ room and parental groups, were discontinued. Although, parents took responsibility for self-care, met at the NICU, and connected outside the unit. Some mothers established maternity groups via Messenger (Facebook), where they could ask questions, debrief, cry together, and support each other. One such group still existed months after discharge, and mothers referred to the group as something they would not be without. *“It is still the mothers in the hospital I ask about things if I have any questions*, *and the father is not in that group”* (Mother F). Hence, the mothers used each other for support during this period.

#### Social media

Parents reported that Messenger, Snapchat, FaceTime, pictures, and films were shared with family, extended family, and close friends. Social media was used to counteract their sadness about not being able to socialize and to show the infant to family and friends. Nevertheless, contact and information to the fathers were prioritized. One mother said that she filmed a situation displaying care in the incubator, filming the entire session. Later she enlarged the film on a computer screen to show the father the infant’s whole body (realistic size), the care, and diaper change.

#### Positive COVID-19 restriction effect

Parents also brought up positive aspects related to the pandemic and COVID-19 restrictions. They appreciated the general decrease in societal infectious diseases. They described an overall social quietness, peace, and ability to solely focus on the infant without welcoming visitors, such as eager family members or friends. Mother R explained: *“*… *then I think the child benefits from more peace and quiet*. *As these infection control measures entailed*.*”* They reported that due to a generally increased compliance with isolation and infection control, there was no need to argue why one could not receive visitors. *"At NICU*, *we also got a peace then*, *that is*, *a peace to be with the child*, *only with the child when I was there with the time we had"* (Mother F). Some fathers expressed that having home office in a way “made up for” the exclusion during the hospitalization, as they had more time to spend with their infant after the hospitalization.

### Struggling to become a family

Parents wanted to be together with their infant; they wanted to explore the new life with the new family member and longed for a feeling of togetherness. One father (Father K) stated: “*It was kind of a difficult start for the whole family; particularly high risk for the father who was somehow disconnected in the beginning*. *Some may be able to recover*, *but not all necessarily”*.

#### Emotional loneliness

Although mothers connected to other mothers at the unit, some sort of emotional loneliness arose through the lack of a present father with whom she could share common experiences and have the possibility of becoming a family.

Because the mothers were the only caregiver with access to the infant for many weeks, she was responsible for informing the father. This was not only in medical aspects, such as weight gain and oxygen fraction, but also as a caretaker for the infant. Mothers felt obligated to give daily reports to the fathers after being in the NICU participating in care and skin-to-skin contact. The fathers could not participate in the infant’s milestones: *“… when the infant smiled for the first time*, *he was annoyed…*..*because then I had experienced it and he did not [laugh and cry*, *father confirms]”* (Mother S).

The lack of a common vocabulary challenged the mothers to explain words, feelings, and medical phenomena, as expressed by one mother (Mother T):

*Yes, we often sat and cried together. While there was a lot, I tried to explain that is difficult to understand… How do you explain the atmosphere inside the NICU? How do you explain that he has had plenty of apneas and was partially gone; that [he] stopped breathing in a minute? How do you explain the alarms? …and the running*?

#### Paternal competency

Later, when the fathers were allowed to visit the NICU, they lined up outside the unit waiting to see their infant. Time was limited. Fathers described how they strived to achieve competence and skills. Mothers carried the responsibility to ensure that the fathers learned from the nurses to care for their preterm and/or ill infant. Fathers were given assignments of what they had to learn and how they should ask nurses to supervise them in specific tasks. There was so much to learn and so little time. Fathers described how they were afraid of doing the wrong thing and that there was no time for necessarily repetition, as father V said: *“no time for repetition learning”*, and *“the learning curve has to be very steep”* (Mother Q).

Mothers reported a significant burden related to carrying the responsibility for the fathers’ learning.

*How did it come to that it was allowed for fathers to be with the mothers but not their infants*? *It is tough to have a premature infant*, *and being the only parent with access*, *or being the one who is not allowed to have a father who feels that he is not a father [crying]*. *It was really tough*! *All the questions and things he was wondering*. *Such practical things with the infant’s development*, *but also how to know the infant; feeling safe and not feeling scared every time he visited the infant*…. *We were never allowed to be there together*… *We could not accomplish any safety together*…. *It has been hard that I know more than him; that I must teach him in everything*. *I must retell everything*, *that we are not equal in a way*. (Mother F)

#### Postponed attachment

Parents reported delayed or a different infant attachment, *“yes*, *we got a different attachment”* (Mother D). The fathers were absent, and due to the tight schedule, mothers were occupied throughout the day. There was limited time to perform skin-to-skin contact and be together with the infant compared to non-pandemic times.

Several fathers described a lack of paternal feelings until discharge and how they gradually got to know their infant for the first time at home; in so doing, it also dawned on them what they have lost along the way. Father K reflected:

*“… You do not get a relationship with the infant in any way. I have children from before. But I saw some of the younger fresh fathers…. they looked completely disconnected”*.

Mother L stated: *“He* [the father] *says that he did not become a father until we came home because he was not allowed to be with his child*. *And it was very strange to think about because [by] then the child was over 2 months old”*.

Mother Q elaborates:

*I never saw my partner as a father. We did not see him as a father until we finished at the hospital… So seeing your child together as parents, I think that is quite important. Standing together to watch "now he does this or that" or "take and change the diaper now" [but] do such things together as one might do normally. Then both parents would be with the child at the same time and do things together and… While now it was like… very set times for when we could come in due to the doctor’s visit very strictly. We could not be there when the shift changed, not be there, and plus we were only one at a time. I was also completely alone in the beginning, and it was like when he was most critically ill. So, I kind of think, I think it has something to do with the delayed attachment, I think*.

The use of social media could not replace parents’ longing for sharing everyday experiences concerning the infant. They were not able to share the same mental pictures of the infant, share joy and concerns. This yearning was related to both the emotional aspects and physical sharing such as looking at the infant and getting to know the infant together. It felt challenging trying to develop an experience to be a family without being together and acting and supporting each other. Being present with the infant caused sorrow and frustration for parents and affected the family dynamic beyond the NICU hospitalization. Mother G said, *“*…*we tried so hard*, *send pictures and keep each other updated*, *but we were in crisis and did not really have the strength for it*… *It would have been nice to let go of that stress*.*”* As father M furthered: *“It’s hard not to see your infant together with the mother*. *In fact*, *it was quite difficult* [crying]”.

Families with several children reported siblings feeling left out by not seeing their new sister or brother for months. This was also a significant additional burden on parents: *“He had home-schooling the whole time we were hospitalized; everything was different for him too*, *and not being able to be there for him…It was almost the worst because I did not know the infant yet …* [crying]” (Mother B). After weeks of hospitalization, a mother met her young son and mother outside the hospital; she felt disobedient to the regulations, but happy.

## Discussion

To our knowledge, this study presents the first in-depth interviews of parents’ experiences with COVID-19 pandemic regulations while hospitalized with a preterm and/or sick newborn infant. Health authorities, hospital administration, and health personal in the NICU were all working in unknown territory under the pandemic. Regulations and information to staff and parents were ground-breaking, as there was no blueprint or obvious right way to handle this new situation. These results reveal that the regulations have strongly impacted parental experiences during NICU hospitalization.

### Life-impacting COVID-19 regulations

Parents in this study were hospitalized during the first wave of the pandemic in 2020. COVID-19 regulations impacted the family’s possibility to visit their infants. During this first wave, a decrease in parental visitation by nearly half was found [11]. Fathers are considered important support for the mothers by virtue of them becoming parents and having an important role in the infant’s future life [21]. At the beginning of the pandemic, there was a general acceptance of the ban on fathers in the units. As time passed and knowledge about the risk of infection transmission increased, the greatest source of frustration among the interviewed parents was related to a lack of consistent information and the closed-door policy for fathers.

Parents reported that nurses, to some extent, shared their frustration on the lack of and inconsistent information about the regulations. This is in line with research that shows how health providers experience increased stress and report a lack of clear and sufficient information in maternal and newborn care during the pandemic [22]. The explanation may be that the information given through the nurses was inconsistent. This proved rather frustrating to parents.

Later, the fathers were allowed to visit their infants two evenings per week. The interviewed fathers described how they were relieved and longed to see their infants. However, after a while, the feeling changed to frustration for some families. They questioned how infection control allowed for visitations on only two evenings when they could live together with the mothers on a daily basis.

Parents wanted clear and thorough information and expected the unit’s leadership to step up and convey this information regularly, both in written and oral forms. Informing families of intrusive restrictions impacting their lives should be a responsibility of the hospital or unit leadership [23]. Presenting equal information to all could probably have prevented some parents from having a feeling that those who shouted the loudest got the most.

There is still sparse knowledge on how fathers´ increased involvement contributes to and affects the family [24]. However, our study’s results showed that fathers’ direct involvement and presence in the NICU were of importance for hospitalized families. A closed-door policy for NICU fathers caused them to miss out on direct contact and significant information, as well as the potential loss of emotional support from medical experts. A concern of insufficient information was reported [7, 23]. The use of virtual technology, e.g., during doctors’ rounds, was recommended [25], but only one father reported having a FaceTime call with a doctor.

### Breastfeeding

Maintenance of pumping and support for early breastfeeding is recommended during the COVID-19 pandemic [26]. The interviewed mothers expressed how the regulation contributed to considerable stress due to the burden of the strict schedule. Not being able to use the breast pump in the unit took time away from being with the infant. Previous research has documented the decreased availability of lactation consultants during the pandemic, restriction of donor milk utilization, and a decrease in breastfeeding rates [9]. In contrast, Malhotra et al. found breastfeeding to increase by hospitalized COVID-19-positive mothers [27]. One may speculate whether isolation and more peace and quiet could positively contribute when establishing breastfeeding. Some mothers in the present study also expressed that the regulation, with fewer people around, gave a sort of quietness and privacy which they appreciated. However, in retrospect, several of them reflected on how they strongly wanted an early discharge. This may have contributed to fewer patients practicing breastfeeding, as starting bottle-feeding meant them going home sooner.

### “It’s intense”—Times out of the ordinary

Due to the pandemic, most of the mothers had already been in prenatal isolation in an obstetric ward due to pregnancy complications or involuntarily self-imposed isolation at home even before their NICU hospitalization. The mothers clearly had a distinct need to bond with other people based on a need for human contact. They sought alliances with others through the mutual understanding of having an infant admitted to a NICU.

Hence, the lack of social contact may have made the mothers more vulnerable and dependent on the mother-nurse relationship. Mothers bonded with the nurses and thereby felt taken care of in an empathic way. Health caretakers’ ability to empathically communicate and involve is known to reduce parental stress more than relaying huge amounts of information [28].

Generally, the Pandemic restrictions made it difficult to interact with peers [29]. Being together with other parents and sharing common experiences has been shown to meet parents’ emotional needs and help them cope during these extremely unordinary times [30]; additionally, peer groups have proved to constitute a useful measure for NICU parents [31]. As NICUs discontinued organizing parent support groups and closed parent rooms, some mothers organized their own support groups. This became an important part of coping with the situation and gave strength to keep up moral both during the NICU stay and after returning home, but also to initiate friendship. This parent-initiated measure worked as psychosocial support and probably also through an educational aspect, which met both effective core elements in structured interventions [32]. Here, there was no input from professionals. It could have been advantageous if health personnel contributed to educational and professional input to increase knowledge and support the parents (e.g., through telemedicine approach) while keeping with the COVID-19 regulation measures [33].

The use of social media was experienced as important by the mothers. This was only occasionally used in communication with staff, but the mothers used it to stay connected with family, friends, and each other. Social media networks strengthened social bonding and created a feeling of connectedness and, hence, may have prevented loneliness after the hospital stay.

The fathers either had limited or no access to the NICU, and never together with the mother. Psychological emotions of suffering, such as anger and sadness, may arise in such extreme situations; also, relational suffering may exist because of the restrictions [34]. This is in line with the experiences of the parents in our study. In addition, the parents did not develop a common language to describe the infant’s condition, behavior, growth, or acute events. They were deprived of an opportunity to share the daily care of the infant. This led to feelings of loneliness. How could the mother explain apnea, where the infant might look dead; explain how it diminished and the extreme fear for the baby when the father had never seen it or been there? Of importance is the mother’s effort and responsibility to inform the fathers in all aspects. How could the mother explain how an infant of, for example, 600 grams looks like in order to counteract possible paternal disconnection from the infant and its experiences and promote attachment? The mothers used pictures and films of the infant in the NICU to show and include the father in his infant’s life. We conclude that filming may help the absent parent to feel more involved in the infant [33].

In the NICU, fathers can feel like outsiders and feel challenged to find their own way to act and fulfill their roles as fathers [35]. This assumption has possibly been strengthened during the COVID-19 restrictions and applied to the fathers in this study, especially with limited access as their visitations became rather busy. In order to prepare for homecoming with their families, they needed to learn infant care skills and receive guided training from the nurses. With limited time, many fathers experienced this as stressful. For mothers who felt responsible for supplementing fathers learning without being present at the NICU, this also led to additional stress.

The COVID-19 restrictions have shown that breastfeeding mothers benefited from staying at and working from home [36]. Our study showed that some fathers benefited from the possibility of home-office and thereby had more time to spend with their new families.

Visiting restrictions are widely implemented in numerous NICUs and have been shown to affect parental presence in general [9]. Essentially, the parents experienced stress and grief over the restrictions and not being able to be together with their infant in the NICU. The fathers did not see their infants for up to 2 months but supported the mothers as best they could. Mothers had a tight and strenuous schedule; in addition, the infant might have a life-threatening illness/situation. In this space, they should develop an attachment to the infant, joint parenting, and nursing skills.

Parents stated that the restrictions affected their bonding to and attachment with the infant. Parent-infant attachment is essential for infant development. Evidence points to the avoidance of separation [37] and that parents’ presence and involvement benefit both infants and their families [38]. The presence and involvement of parents may ameliorate their infants’ neuroendocrine stress responses, accordingly mitigating or even preventing toxic stress, and may influence long-term outcomes [37–40].

In order to support parents and infants admitted to NICUs during the COVID-19 pandemic, the importance of thorough and consistent information cannot be overemphasized. Parents also need to be supported both practically and psychologically to reduce any feelings of alienation and help to build a strong, nuclear family. Parent support groups and parent peer groups should probably be continued, if not physically then digitally.

## Conclusion

Health authorities, hospital administration, and health personal in NICUs have been working in unknown territory during the current pandemic. This study grants a glimpse into how some parents experienced their stay in the NICU during the first wave of the COVID-19 pandemic. The regulations had strong negative impact on parental experiences during the NICU hospitalization. The restriction to fathers’ access to the NICU acted as a significant obstacle to early infant-father bonding and led to loneliness and isolation by the mothers. Thus, these COVID-19 measures might have had adverse consequences for families.

### Strength and limitations

Strength of this study was the attempt to capture how families hospitalized in the NICU experience the COVID-19 measures. This study was originally initiated by the two user organizations to provide new knowledge on the user’s experiences.

Health authorities are encouraged to include users’ voice and experiences in knowledge-based practice. To explore and capture parental consequences of the COVID-19 regulations a qualitative approach is well suited, as quantitative epidemiological studies may miss out the social implications [41].

Parents were free to participate in the interviews either alone or as a couple. One may speculate if both parents were interviewed either alone or together consequently, and that this would lead to more focused data corpus. At the other hand it is important to secure that all participants feel well in the interview situation.

We had to align with the actual COVID-19 regulations in the society. Therefore, we performed both distancing in face-to-face interviews, and digital interviews, this also as the choice of the parents. Conducting in-deep interview “on distance” on the web is a new situation for most researchers, which demand critical assessment regarding privacy regulations and on how interviewing on distance might have impact on the interview situation [42]. Our experiences with the secured JOIN application were good, but parents may feel detached, but at the other hand this distance may make parents feel comfortable.

Generally, the parents appreciated the possibility to tell their story.

## Supporting information

S1 File Semi-structured interview guide. (DOCX)

S2 File COREQ checklist. (DOCX)
