# Supplementary material for: Starting parenting in isolation a qualitative user-initiated study of parents’ experiences with hospitalization in Neonatal Intensive Care units during the COVID-19 pandemic
Source: PLoS One. 2021 Oct 29;16(10):e0258358. doi: 10.1371/journal.pone.0258358 (PMC8555791; doi:10.1371/journal.pone.0258358)
Supplement: S1 File — (DOCX) [file pone.0258358.s001.docx]

Interview guide - semi-structured

The guide is divided into topics we want to talk to the parents about, sub-questions are used to support interviews. The focus is on what the parents say about their experiences.

# Demographic data

The parents tell about their infant and themselves.

Parents tell about how, why and how long their infant was hospitalized

# Topic 1: About the hospital stay

The parents talk about the visitation regulations and how they experienced them

# Topic 2: Parents' experiences of care

The parents talk about how they experienced infant care and care for eventually siblings and for themselves

Parents talk about how to be a parent in this period and how they managed this period.

# Topic 3: Return home or transfer to another hospital

Parents talk about how they were prepared to come home after hospitalisation

Parents talk about how they experienced to come home after hospitalisation
