# Supplementary material for: Starting parenting in isolation a qualitative user-initiated study of parents’ experiences with hospitalization in Neonatal Intensive Care units during the COVID-19 pandemic
Source: PLoS One. 2021 Oct 29;16(10):e0258358. doi: 10.1371/journal.pone.0258358 (PMC8555791; doi:10.1371/journal.pone.0258358)
Supplement: S2 File — (DOCX) [file pone.0258358.s002.docx]

# S1 File: COREQ 32-item checklist

Tong A, Sainsbury P, Craig, J. Consolidated criteria for reporting qualitative research (COREQ): a 32-item checklist for interviews and focus groups. Int J Qual Health Care. 2001;19(6): 349-357. doi:10.1093/intqhc/mzm042.

| **No.** | **Item** | **Guide**  **questions/descriptions** | **Reported on page** |
| --- | --- | --- | --- |
|  | Domain1:  Research team and reflexivity | | |
|  | **Personal Characteristics** | | |
| 1. | Interviewers/facilitators | Which author/s conducted the interview or focus group? | The first (NMK) and the last (BST) author conducted the interviews. |
| 2. | Credentials | What were the researcher’s credentials? | NMK is a pediatric and critical care RN and holds a Ph.D.; BST is an RN and holds a Ph.D. Drude Fugelseth is a Professor at the Faculty of Medicine, University of Oslo & Consultant Neonatology at Oslo University Hospital, Oslo Area, Norway. LMMK is a pediatric RN and holds an MNSc. |
| 3. | Occupation | What was their occupation at the time of the study? | NMK works as an associate professor at Oslo Metropolitan University and is an associate at OUH, Department of Neonatal Intensive Care Unit. BST works as a neonatal nurse and researcher at Viken Hospital Trust. She is also an associate at OUH, Department of Neonatal Intensive Care Unit, and an associate professor at Lovisenberg Diaconal University College. DF works as a researcher and consultant in neonatology. LMMK works as a consultant in the Norwegian Neonatal Network. |
| 4. | Gender | Was the researcher male or female? | All researchers are female. |
| 5. | Experience and training | What experience or training did the researcher have? | The first author is an experienced qualitative researcher, and BST has extensive experience in performing interviews as a research method. DF has extensive experience in research in the field of neonatology. LMMK has former experience with qualitative study designs and facilitates data for research in the field of neonatology in her daily work. |
|  | **Relationship with participants** | | |
| 6. | Relationship established | Was a relationship established prior to study commencement? | No, the researchers did not know or meet any of the participants. They did not work in the clinical department at the time. |
| 7. | Participant knowledge of the interviewer | What did the participants know about the researcher/s? | A letter to possible participants informed about the study’s aim, design, and research questions. It also informed participants that the Norwegian Premature Association and the Norwegian Association for Children with Congenital Heart Disease initiated the study. |
| 8. | Interviewer characteristics | What characteristics were reported about the interviewer/facilitator? | Participants were informed about the researchers’ credentials, occupations, and clinical experience; additionally, how the interviews were planned. |
|  | Domain 2:  study design | | |
|  | **Theoretical framework** | | |
| 9. | Methodological orientation and Theory | What methodological orientation was stated to underpin the study? | We used thematical analysis. |
|  | **Participant selection** | | |
| 10. | Sampling | How were participants selected? | Parents who had been admitted to the NICU for >13 days during the most comprehensive restrictions. |
| 11. | Method of approach | How were participants approached? | Through letters by national mail. A total of 47 families were eligible for inclusion and were approached by information letters. They were screened according to inclusion and exclusion criteria through The Norwegian Neonatal Network. They contacted the researchers if they were interested in hearing more about the study or wanted to participate. |
| 12. | Sample size | How many participants were in the study? | Nine mothers and four fathers. |
| 13. | Non‐participation | How many people refused to participate or dropped out? Reasons? | Families that did not want to participate did not approach the researchers. We do not have any information about them. |
|  | **Setting** | | |
| 14. | Setting of data collection | Where was the data collected? | Data were collected either in a quiet room at a university college or through a secure web platform (join.nhn.no) from Norsk Helsenett, owned by the Norwegian Ministry of Health and Care Services. |
| 15. | Presence of non-participants | Was anyone else present besides the participants and researchers? | Only NMK, BST, and the participants. |
| 16. | Description of sample | What are the important characteristics of the sample? | They were parents of preterm and/or sick infants admitted to either NICU only or the Department of Heart Surgery and the NICU. |
|  | **Data collection** | | |
| 17. | Interview guide | Were questions, prompts, guides provided by the authors? Was it pilot-tested? | Yes, we used a semi-structured interview guide (Supplementary fil 1)).  The interview was not pilot-tested with parents. |
| 18. | Repeat interviews | Were repeat interviews carried out? | No |
| 19. | Audio/visual recording | Did the research use audio or visual recordings to collect the data? | An audio recorder was used. |
| 20. | Fieldnotes | Were field notes made during and/or after the interview? | Fieldnotes were made during the interviews, and NMK and BST discussed the notes after finishing the interviews. |
| 21. | Duration | What was the duration of the interviews | 34–65 minutes |
| 22. | Data saturation | Was data saturation discussed? | Yes |
| 23. | Transcripts returned | Were transcripts returned to participants for comment and/or correction? | No |
|  | Domain 3:  analysis and findings | | |
|  | **Data analysis** | | |
| 24. | Number of data coders | How many data coders coded the data? | NMK & BST |
| 25. | Description of the coding tree | Did the authors provide a description of the coding tree? | Yes, Figure 1. **Map with the final overarching themes and the belonging sub-themes** |
| 26. | Derivation of themes | Were themes identified in advance or derived from the data? | They were derived from the data – inductive. |
| 27. | Software | What software, if applicable, was used to manage the data? | None |
| 28. | Participant checking | Did participants provide feedback on the findings? | No |
|  | **Reporting** | | |
| 29. | Quotations presented | Were participant quotations presented to illustrate the themes/findings? Was each quotation identified? | Yes |
| 30. | Data and findings consistent | Was there consistency between the data presented and the findings? | Yes |
| 31. | Clarity of major themes | Were major themes clearly presented in the findings? | Yes |
| 32. | Clarity of minor themes | Is there a description of diverse cases or discussion of minor themes? | Yes |
